# Iterative refinement of structure-based sequence alignments by Seed Extension

**DOI:** 10.1186/1471-2105-10-210

**Published:** 2009-07-09

**Authors:** Changhoon Kim, Chin-Hsien Tai, Byungkook Lee

**Affiliations:** 1 Laboratory of Molecular Biology, Center for Cancer Research, National Cancer Institute, National Institutes of Health, Bethesda, Maryland 20892, USA

## Abstract

**Background:**

Accurate sequence alignment is required in many bioinformatics applications but, when sequence similarity is low, it is difficult to obtain accurate alignments based on sequence similarity alone. The accuracy improves when the structures are available, but current structure-based sequence alignment procedures still mis-align substantial numbers of residues. In order to correct such errors, we previously explored the possibility of replacing the residue-based dynamic programming algorithm in structure alignment procedures with the Seed Extension algorithm, which does not use a gap penalty. Here, we describe a new procedure called RSE (Refinement with Seed Extension) that iteratively refines a structure-based sequence alignment.

**Results:**

RSE uses SE (Seed Extension) in its core, which is an algorithm that we reported recently for obtaining a sequence alignment from two superimposed structures. The RSE procedure was evaluated by comparing the correctly aligned fractions of residues before and after the refinement of the structure-based sequence alignments produced by popular programs. CE, DaliLite, FAST, LOCK2, MATRAS, MATT, TM-align, SHEBA and VAST were included in this analysis and the NCBI's CDD root node set was used as the reference alignments. RSE improved the average accuracy of sequence alignments for all programs tested when no shift error was allowed. The amount of improvement varied depending on the program. The average improvements were small for DaliLite and MATRAS but about 5% for CE and VAST. More substantial improvements have been seen in many individual cases. The additional computation times required for the refinements were negligible compared to the times taken by the structure alignment programs.

**Conclusion:**

RSE is a computationally inexpensive way of improving the accuracy of a structure-based sequence alignment. It can be used as a standalone procedure following a regular structure-based sequence alignment or to replace the traditional iterative refinement procedures based on residue-level dynamic programming algorithm in many structure alignment programs.

## Background

In searching for protein functions and in building homology models, it is desirable to have accurate sequence motifs and profiles [1-3], which are obtained from sequence alignments of homologous proteins. However, it is often difficult to obtain accurate sequence alignments based on sequence similarity alone when sequence similarity is low.

Therefore, structural alignments, when available, have been used to guide sequence alignments. Such structure-based sequence alignments have been used as the gold standard to evaluate pure sequence alignment methods [4,5] and to derive structural environment-specific substitution matrices which have been shown to be useful for detection of remote homologs and for sequence-structure alignments [6-9].

However, structure-based sequence alignments produced by different programs can be different even when the structures are similar [10,11]. There are a large number of instances wherein all or parts of the structure are shifted by 2 or 4 residues or even by an odd number of residues [12]. Some methods are probably quite good at detecting structural similarity, yet relatively poor in terms of the accuracy of the sequence alignment they produce [12].

DaliLite and VAST use a Monte-Carlo procedure after initial structural alignment [13,14], FATCAT and MATT adopt AFP (aligned fragment pair)-based dynamic programming without constructing initial structural alignments [15,16], and other programs mostly rely on residue-level dynamic programming algorithm according to various scoring schemes with or without initial rigid-body superposition [17-20].

We previously developed the SE (Seed Extension) algorithm which generates a sequence alignment from a superimposed structure pair without changing the superposition [21]. A number of other programs [22-25] also provide a similar function, but these programs use the dynamic programming algorithm and a gap penalty. We have shown that SE, which is not based on the dynamic programming algorithm and does not use a gap penalty, generates a more accurate alignment on average than programs that use a dynamic programming algorithm.

In this study, we report on the development of a fast refinement procedure, which can be used to improve an existing structure-based sequence alignment. The procedure, which we call RSE (Refinement with SE), is an iterative procedure that uses SE in its core. Using CDD (Conserved Domain Database) [26] "root node set" as the reference alignment [12], we show that appending the RSE procedure to a structure-based sequence alignment program improves the accuracy of the alignment for all 9 programs tested.

## Results

### Improvement of the overall alignment accuracy

In order to see if the RSE procedure improves or degrades alignments produced by different structure comparison programs, we ran the program to be tested with default options to obtain the structure-based sequence alignment for each structure pair. Then the sequence alignment and the corresponding structure pair were fed to the RSE program to obtain a new sequence alignment. We used the fraction of correctly aligned residues with shift error δ, f_CAR_(δ), or F_CAR_(δ), which is f_CAR_(δ) averaged over all structure pairs in a superfamily, as the measure of accuracy of the alignment for each superfamily [12]. Since there were 96 superfamilies in the dataset (Table 1), we took the average over all superfamilies, <F_CAR_(δ)> (angle brackets for averaging), as the measure of the overall accuracy of alignments for the whole dataset for a given method.

**Table 1:** Composition of the CDD root node set

SCOP class	Number of CDs^†^	Number of structure pairs
all-α (**a**)	11	326
all-β (**b**)	15	1721
α/β (**c**)	35	912
α+β (**d**)	26	510
others^§^(**o**)	9	122
total (**t**)	96	3591

^§ ^Other than the four major classes.

^† ^The 5 outlier superfamilies were excluded

**Figure 1:**
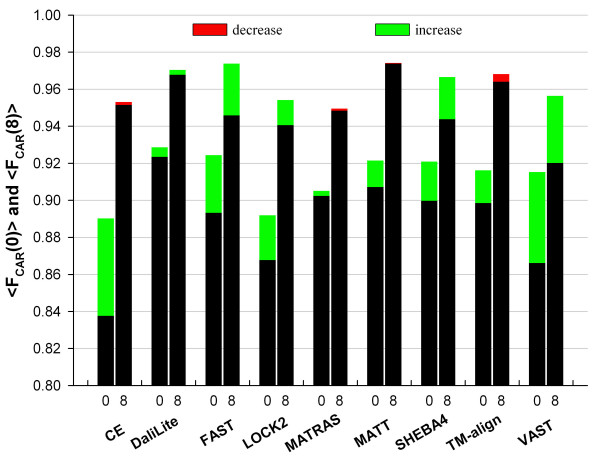
**Average improvements of structure-based sequence alignments**. The average accuracies of sequence alignment were computed for each method before and after refinement by the RSE procedure. The corresponding method name is given under each pair of bars along the x-axis, where the bars for <F_CAR_(0)> and <F_CAR_(8)> are marked with 0 and 8, respectively. The black portion together with the red tip, when present, represent <F_CAR_(0)> or <F_CAR_(8)> before the refinement. The green and red tips indicate the increment and the decrement, respectively, after the refinement.

**Figure 2:**
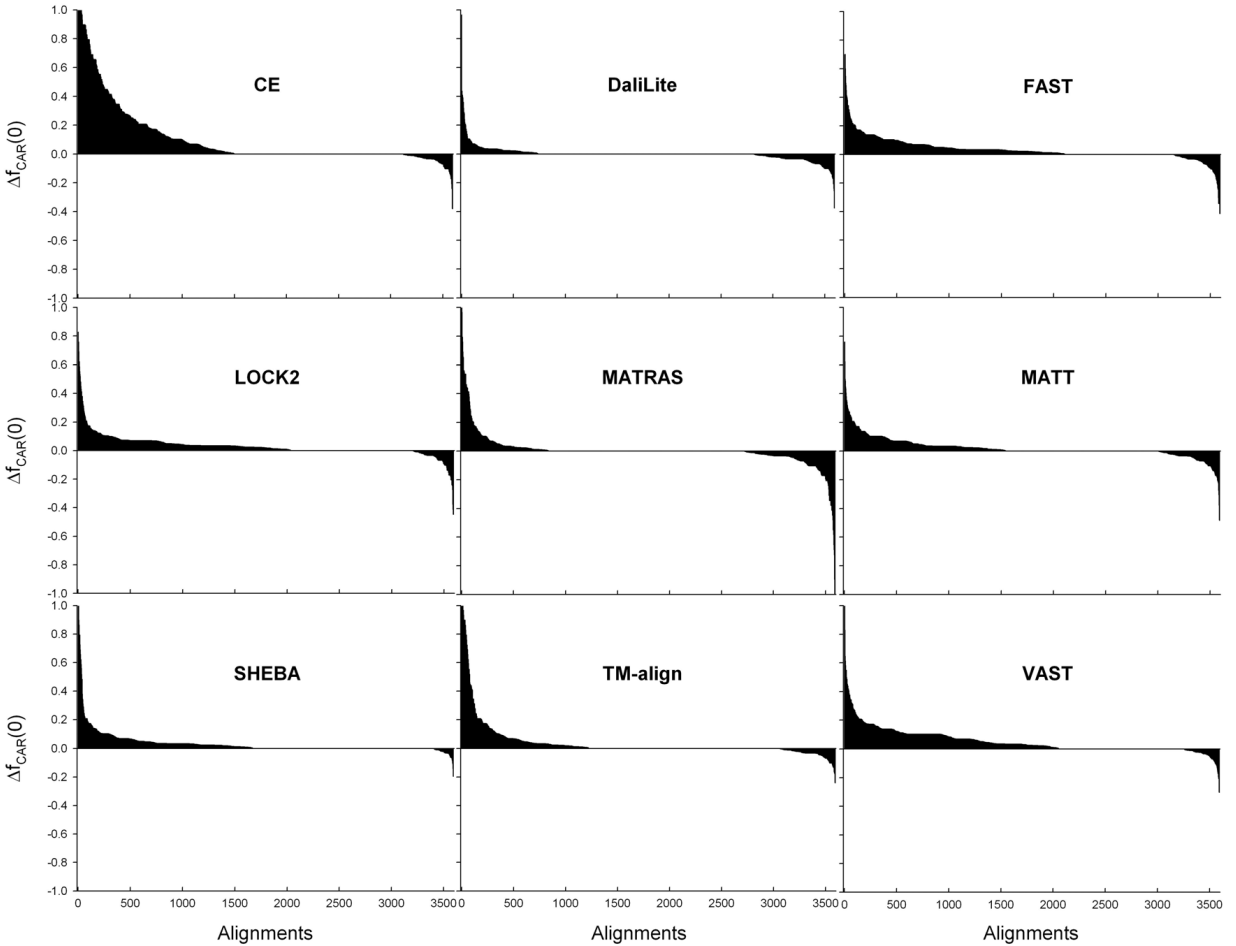
**Change in accuracy of each individual alignment after refinement**. The alignments from each method were sorted according to their Δf_CAR_(0), which is the f_CAR_(0) of the refined alignment minus that of the original alignment. The nine graphs, one for each method, are arranged in alphabetical order. The x- and y- axes in each graph represent the alignment and Δf_CAR_(0), respectively. The number of alignments for which f_CAR_(0) increased and decreased are, respectively, (1495, 469), (733, 770), (2130, 437), (2029, 383), (835, 867), (1542, 581), (1669, 182), (1221, 182), and (2054, 334), for CE, DaliLite, FAST, LOCK2, MATRAS, MATT, SHEBA, TM-align, and VAST, respectively.

RSE procedure improved the alignment accuracy, as measured by <F_CAR_(0)>, for all methods (Figure 1). The improvements were small for DaliLite and MATRAS but about 5% for CE and VAST. The alignments from FAST, LOCK2, and TM-align also improved even though these programs were designed to give high quality sequence alignments [17,18,20]. There were more alignments with accuracy gain than those with accuracy loss, except for DaliLite and MATRAS (Figure 2). The increase in the number of correctly aligned residues is large for many alignments, especially for CE, SHEBA, TM-align, and VAST, while a decrease, when happens, is always relatively small in magnitude, except for a few pairs for MATRAS.

The nature of the improvement varied among different methods. For CE, MATT and TM-align, RSE improved <F_CAR_(0)> but not <F_CAR_(8)> (Figure 1), which indicates that it is mostly alignment shift error that was reduced by the RSE procedure. For FAST and SHEBA-4, the improvements appear to be mainly correction of under-alignments, presumably by reducing the number of gaps, since <F_CAR_(8)> increased almost as much as <F_CAR_(0)> by the refinement.

**Figure 3:**
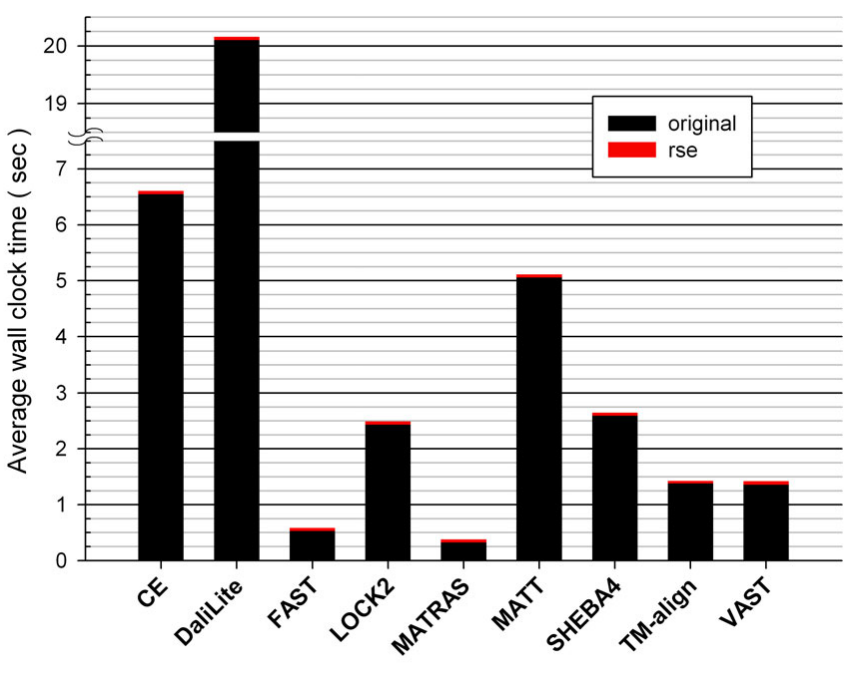
**Average execution times**. The total wall clock times for each method to align 3,591 pairs and for the RSE to refine them were recorded on Dual 2 GHz PowerPC G5 with 4GB memory, running Mac OS X version 10.3.9. The pre-processing times for MATRAS and VAST were not included. The x- and y-axes show the methods and the average times in seconds, respectively. The times taken by the methods and by the RSE are shown in black bars and red tips, respectively.

MATT is a unique method in that it considers the flexibility of structures to improve the sequence alignment quality [16], but its overall accuracy with the root node set was still worse than that of DaliLite and could be noticeably increased by the RSE procedure (Figure 1). The RSE-augmented MATT, FAST, and SHEBA-4 achieved <F_CAR_(0)> values that were now comparable to that of DaliLite, which is a much slower program (Figure 3).

### Computing time

**Figure 4:**
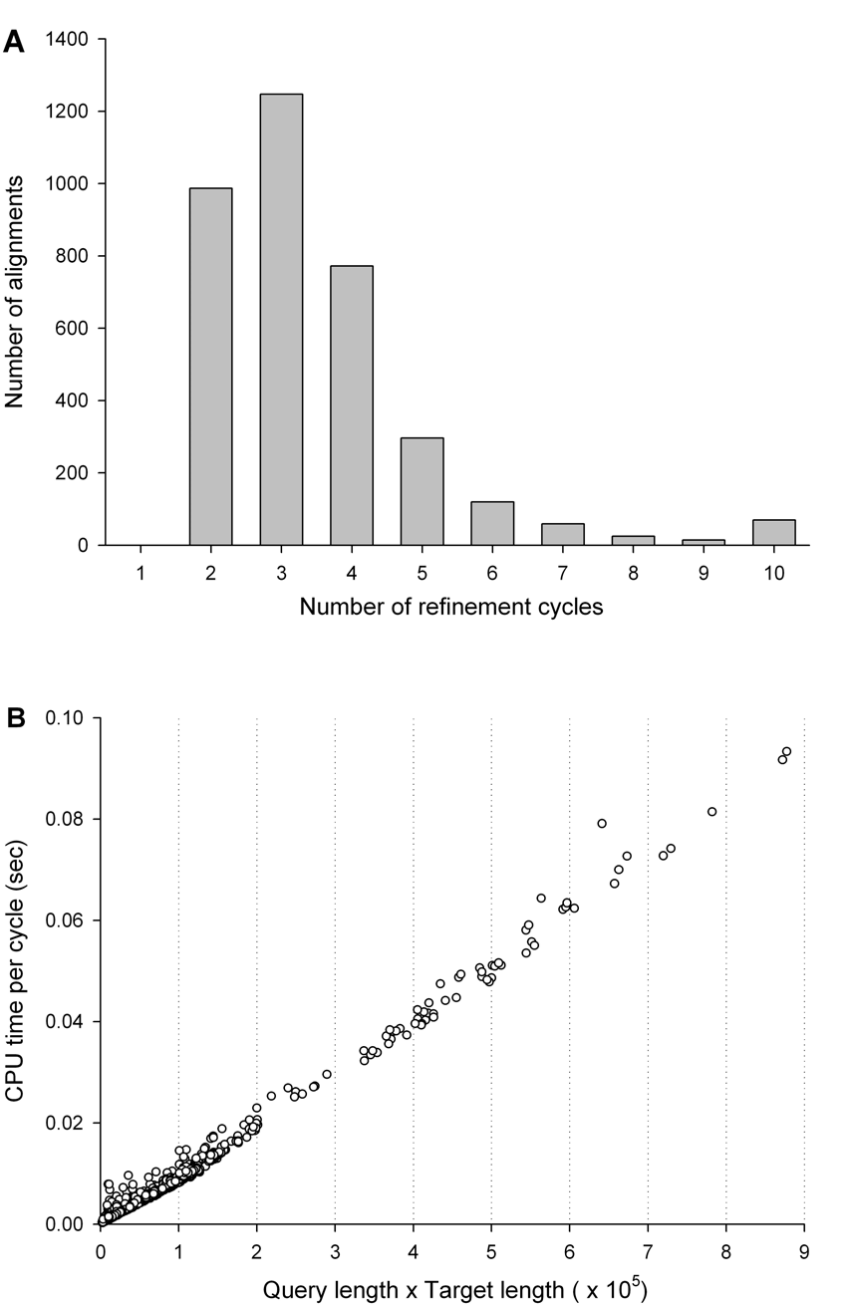
**Time complexity for RSE**. The number of refinement cycles and the CPU time per refinement cycle were recorded for each of the 3,591 CDD alignments. Panel (A) gives the histogram of the number of alignments vs. the number of refinement cycles. There is no alignment with one cycle because RSE always executes one additional final cycle (unless no alignment is found in the first cycle). Panel (B) gives the CPU times per cycle as a function of the size of the alignment matrix.

The times spent by the RSE procedure were nearly negligible compared to the total times spent by the programs to align the structure pairs: RSE took about 46 to 60 milliseconds of wall clock time per alignment on average (Figure 3). In order to measure time complexity for the RSE procedure in terms of CPU times, we focused on the refinement of CDD alignments, since the average wall clock times for all methods were similar (Figure 3). The number of cycles used by RSE to reach the final alignment varied for different structure pairs (Figure 4A) and affected the overall computing time. However, the CPU time per cycle showed linear dependence on the product of the query and target lengths (Figure 4B).

### Dependence on structural types

**Figure 5:**
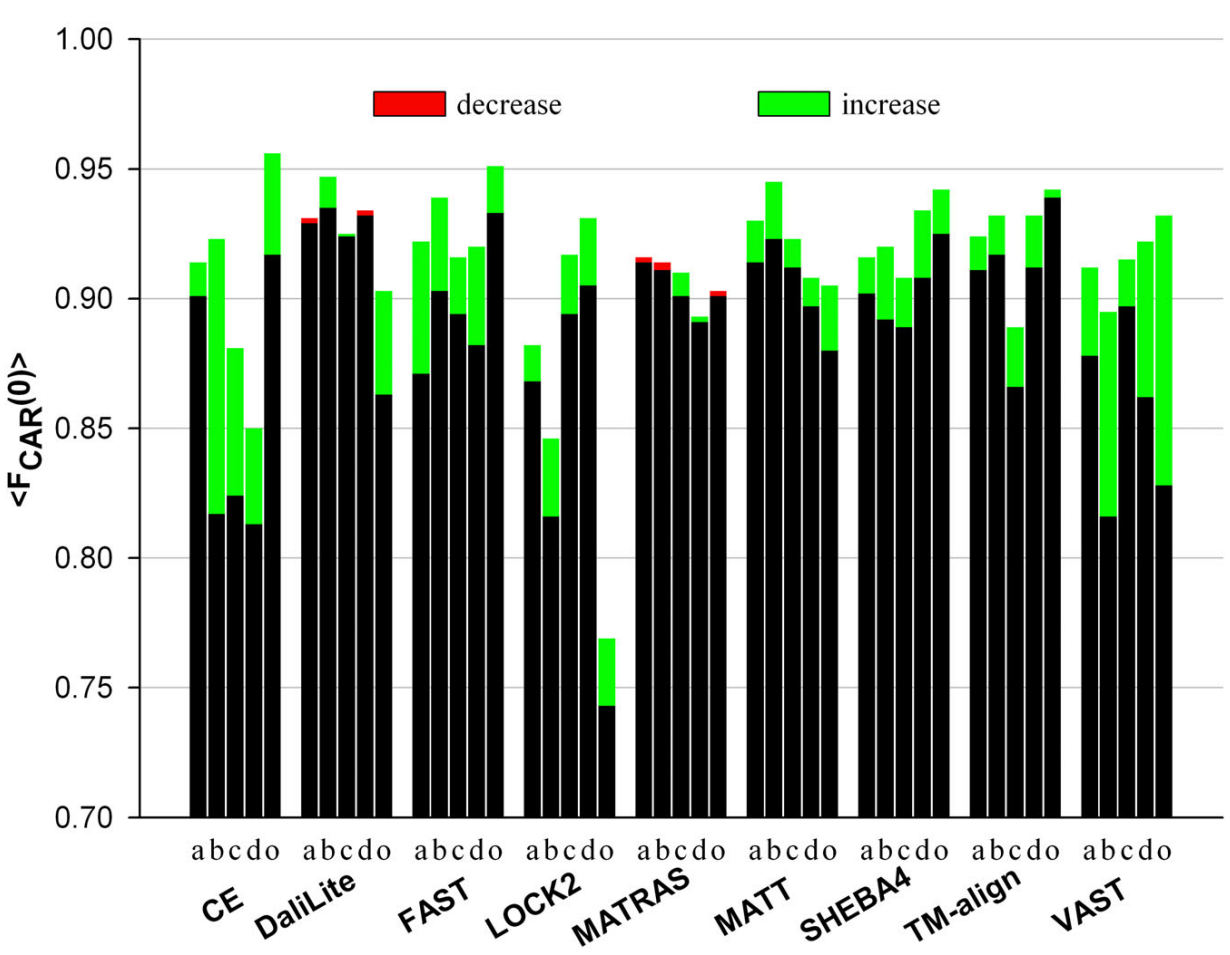
**Dependence of improvement by the RSE procedure on the SCOP class**. The structure pairs were grouped according to their SCOP class and then <F_CAR_(0)> in each class was computed for each method before and after refinement by the RSE procedure. The SCOP class names in single characters are under each bar along the x-axis: **a**, **b**, **c**, and **d **for all-α, all-β, α/β, and α+β classes, respectively; **o **for the other (other than **a **to **d) **classes. Color scheme is the same as in Figure 1.

In order to see if the improvement of the alignment depended on protein structural types, the structure pairs were grouped according to their SCOP class (Table 1) and <F_CAR_(0)>s were computed for each class and method (Figure 5). The RSE procedure improved the <F_CAR_(0)> for most classes (the green tips), but there were cases wherein <F_CAR_(0)> decreased by a small amount (the red tips in the case of DaliLite and MATRAS). The <F_CAR_(0)> increases were most prominent for FAST and VAST across all SCOP classes and for CE for the β-sheet containing classes. The alignments also improved for LOCK2, SHEBA-4, MATT and TM-align for all SCOP classes. The <F_CAR_(0)> in "others" class in DaliLite increased to a comparatively large extent, indicating that certain defects in its alignments were effectively corrected.

### Refinement of good and not-so-good initial input alignments

When RSE was run on the reference CDD alignment, f_CAR_(0) decreased for 1589 out of 3591 pairs, making the <F_CAR_(0)> value to decrease by approximately 5% (Table 2). Since CDD is being used as the standard, any change in alignment will reduce the <F_CAR_(0)> value. However, the <F_CAR_(0)> value remained higher than that from any structure comparison programs (see Table 2 and Figure 1).

**Table 2:** Average performance of the control methods

Methods	CDD+RSE	DaliLite		SSEARCH		SALIGN	
		
		-	+RSE	-	+RSE	-	+RSE
<F_CAR_(0)>	0.946	0.923	0.929	0.471	0.665	0.548	0.748
<F_CAR_(8)>	0.981	0.968	0.970	0.634	0.726	0.664	0.808
<f_CAR_(0)>	0.963	0.928	0.929	0.326	0.494	0.411	0.592
<f_CAR_(8)>	0.988	0.984	0.984	0.507	0.612	0.577	0.793

RSE also improved the accuracy of the alignments from the pure sequence alignment program SSEARCH by 19% to about 67% and from the profile-profile alignment program SALIGN by 20% to 75% (Table 2). This shows that RSE improves even a poor alignment. But the final accuracy attained was substantially lower than those from any structure comparison programs.

### Comparison of improvements between SE and RSE

**Figure 6:**
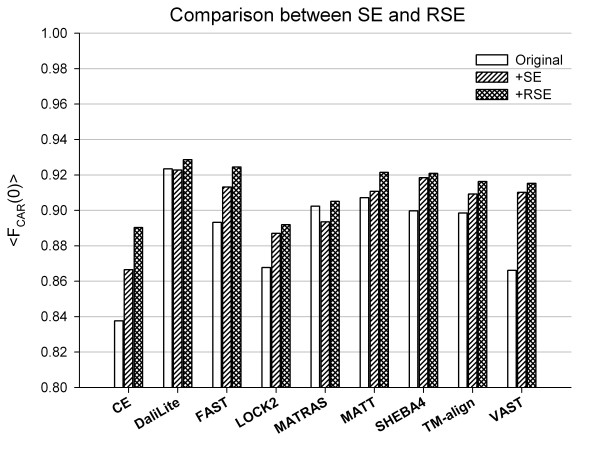
**Average fraction of correctly aligned residues before and after modification by either SE or RSE**. The average accuracies of sequence alignment were computed for each method before (white) and after modification either by SE (hatched) or RSE (crosshatched). The method name is given under each group of three bars along the x-axis. The y-axis gives <F_CAR_(0)>, the average fraction of correctly aligned residues, averaged over the superfamily.

The performance of RSE was compared to that of the original SE (Figure 6). SE produced improved alignments for 7 methods, but poorer alignments for DaliLite and MATRAS. RSE made additional improvements for all methods, although the extent of the improvement varied for different methods.

**Figure 7:**
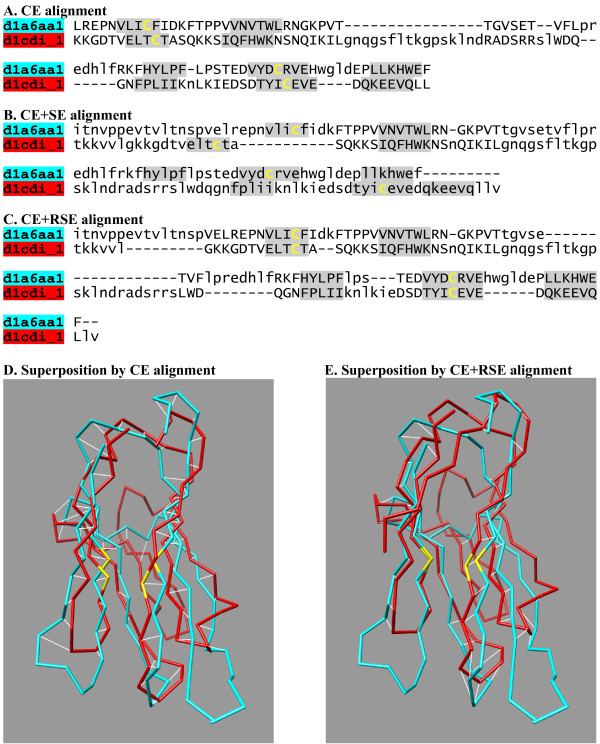
**An example of the refinement of the CE alignment**. The structure-based sequence alignments by CE alone (A), CE followed by SE (B) and CE followed by RSE (C) are shown with the shaded blocks indicating CDD reference alignments. The disulfide-forming cysteine residue pairs are highlighted in yellow. The aligned and unaligned residues are indicated by uppercase and lowercase letters, respectively. The panels D and E show the superpositions by CE and after refinement by RSE, respectively. The aligned residue pairs are indicated by white lines. The cysteine residues are in yellow. The blue and red structures represent the SCOP domains d1a6aa1 and d1cdi_1, respectively.

To give concrete examples of improvement, Table 3 lists pairs in the immunoglobulin superfamily for which RSE made most improvement. It can be noted that many programs generate alignments in which no residue pairs are correctly aligned. Panels A and D in Figure 7 show the sequence alignments and the structural superposition, respectively, produced by CE for the pair given in Table 3. Shaded blocks in the sequence alignments indicate the residues aligned in the CDD reference alignment. Note that all the residues in the shaded blocks in panel A are shifted by one residue, resulting in an out-of-phase superposition of β-strands and the mis-alignment of the signature Cys residues of the immunoglobulin domains (panel D). For the pairs shown in Table 3, DaliLite, CE, MATRAS, SHEBA4, TM-align, and VAST produced sequence alignments with one residue shift, while FAST, MATT, and LOCK2 produced those with some residues shifted by two residues.

**Table 3:** The most improved case in immunoglobulin superfamily (cd00096) for each method

		f_CAR_(0)		
		
Program	Protein pair (SCOP domains)	-	+SE	+RSE
CE	d1a6aa1-d1cdi_1	0.000	0.207	1.000
DaliLite	d1fg2b_-d1wioa2	0.000	0.207	0.966
FAST	d1a1ma1-d2ig2h1	0.345	0.000	0.862
LOCK2	d1c5da1-d1cid_1	0.172	0.414	1.000
MATRAS	d1ev2e2-d1i1ad2	0.000	0.000	1.000
MATT	d1cs6a1-d1f3jb1	0.241	0.138	1.000
SHEBA4	d1a2yb_-d1e4ka2	0.000	0.000	1.000
TM-align	d1f3jb1-d1vcaa2	0.000	0.207	1.000
VAST	d1a2yb_-d1cqka_	0.000	0.000	1.000

RSE could correct these alignments, unlike SE (panels B and C). Since SE just derives a sequence alignment from a given structural superposition without changing it, it cannot correct a bad superposition. In contrast, RSE iteratively adjusts the structural superposition, which can result in a large improvement.

### Quality of the CDD alignments as the standard

In order to better understand the nature of the changes of the CDD alignments by the RSE procedure, many cases were visually inspected. There were 136 pairs (3.4% of all pairs) from 21 different superfamilies for which the f_CAR_(0) in RSE-refined CDD alignment decreased by more than 20%. As expected, some of these structure pairs were from the cd00531 (7 pairs) and cd01984 (6 pairs) superfamilies, for which our previous study [12] indicated that the CDD alignments were in error. For some pairs from two other superfamilies (cd00198 and cd00385), RSE again appeared to produce more reasonable alignments than CDD, in terms of the distances and orientations of side chains between aligned residues. Fourteen pairs including the worst three cases were from cd00688, which are made of α/α toroid structures (a barrel made of two layers of alpha-helices). Not all helices in these structures could be superposed simultaneously without ambiguity and RSE produced tilted alignments. There were 47 pairs from the three superfamilies having the (β/α)_8 _TIM-barrel structure (cd01292, cd00415 and cd00945), for which the inner layer of beta-strands were reasonably alignable but the outer helices were not. There were other helix-containing superfamilies (cd00389, cd00397, cd00198, and cd00385), for which at least one pair of alpha-helices was not unambiguously alignable. For some pairs in cd00158, CDD has pairs of residues aligned, which RSE could not align because they were too far apart from each other in an irregularly shaped region of the superposed structures. These were aligned in CDD presumably by sequence similarity.

## Discussion

Structure-based sequence alignments are not as robust as one would like. In some cases, they can be inherently ambiguous. But more frequently different structure alignment programs generate alignments that contain errors that can be easily recognized by human experts. We showed in a previous study [12] that, the overall average accuracy of structure-based sequence alignments, as measured by <F_CAR_(0)> with the CDD root node set as the reference, ranged from 81% to 89% depending on the program used. When the five outlier superfamilies [12] are excluded, it ranges from 84% to 92% (Figure 1). The two newly included methods, TM-align [20] and MATT [16], are not exceptional in this regard.

The RSE procedure reported here was designed to improve the structure-based sequence alignments. It uses the previously reported SE algorithm [21] to obtain a refined sequence alignment from an input alignment. SE is a heuristic algorithm that produces an alignment from two superimposed structures without using a gap penalty. Figure 1 shows that the average accuracy improved for all structure alignment programs tested by adding the RSE refinement procedure. Notably, alignments from MATT, which is a program that considers structural flexibility, could also be improved significantly by the RSE procedure, which does not explicitly consider structural flexibility. RSE reduced the shift error for most programs since the refinement increases F_CAR_(0) more than F_CAR_(8). For FAST and SHEBA-4, RSE seems to lengthen the alignment also since F_CAR_(8) and F_CAR_(0) increased to a similar extent. The alignments improved for structure pairs from all SCOP classes for most of the programs tested (Figure 5).

Impressively, the alignments from FAST, one of the fastest programs, could be improved to about the same level of accuracy as those from DaliLite, the best performer without RSE (Figure 1). The accuracies of MATT and SHEBA-4 also increased to similar levels. These improvements were achieved with nearly negligible increase in overall processing times (Figures 2 and 3). Therefore structure alignments can be done with substantially reduced computational cost without compromising accuracy by combining RSE with one of the fastest programs. Alternatively, the RSE procedure can be implemented to replace the traditional residue-based dynamic programming algorithm in a structure comparison program that uses it to improve both the accuracy and computing time.

An ideal refinement procedure will fix incorrectly aligned regions without degrading the correctly aligned ones (Figure 7). Unfortunately, RSE seems to degrade some alignments when compared to the CDD alignments (Figure 2). When the CDD alignment itself was used as the initial alignment for an RSE procedure, <F_CAR_(0)> and <f_CAR_(0)> decreased to about 95% and 96%, respectively (Table 2). According to our visual inspection of a number of cases for which f_CAR_(0) fell to a value below 80%, the RSE procedure appears to have found an alternate alignment or to have corrected CDD errors in most cases. We expect that similar causes are at work for at least some of the cases seen in Figure 2 for which there is an apparent degradation of alignment accuracy.

RSE greatly improves the alignments from SSEARCH and SALIGN, which are non-structure-based, pure sequence-based alignment procedures (Table 2). This is to be expected since use of the structural information should improve the sequence alignment. One notes, however, that the average accuracy attained after the refinement is far below those of any of the structure alignment methods (Compare the numbers in Table 2 and the bar heights in Figure 1). This indicates that the outcome of the RSE procedure does depend on the quality of the input alignment. One can also note that there are about 7 to 11% error left after the RSE refinement of the alignments of all methods (Figure 1) and that no method reached the accuracy of refined CDD alignments (about 95% in Table 2). These observations imply that RSE could not correct certain errors of the input alignments. This could happen because some needed seed alignments could not be found from a poor initial superimposed structures and/or because of the constraints imposed by the inflexible, rigid body superposition of structures.

## Conclusion

We devised a refinement procedure for structure-based sequence alignments, called RSE. It uses the SE algorithm, which produces a sequence alignment without using a gap penalty. When applied to the structure-based sequence alignments generated by various structure comparison/alignment programs, the average accuracy increased for all programs tested. This refinement procedure is fast enough to be routinely used as a supplemental procedure following a regular structure-based sequence alignment or to replace the traditional dynamic programming algorithm-based refinement procedure which is a part of many structural alignment programs.

## Methods

### The RSE procedure

We first briefly describe the SE algorithm [21]. Given a pair of superimposed structures A of length *m *and B of length *n*, define two *m *× *n *matrices *M *and *SP*. *M *is the matrix of average C_α _distances defined as , where *d*_*ij *_is the distance between the C_α _atoms of residue *i *of structure A and residue *j *of structure B. *SP *is the matrix of scalar products; *SP*_*ij*_, is the scalar product between two unit vectors which bisect the angles formed by three consecutive C_α _atoms, (i-1, i, i+1) for structure A and (j-1, j, j+1) for structure B. A pair of residues (*i, j*) is a seed if its corresponding matrix element *M*_*ij *_is the minimum in both the *i*^*th *^row and the *j*^*th *^column of the matrix and *SP*_*ij *_is greater than 0. A set of consecutive (non-gapped) seeds defines a seed segment. The SE algorithm consists of the following steps:

1. Find *seeds *and *seed segments*.

2. Find *aligned segments *by extending *seed segments*.

3. Find the consistent set of *aligned segments *with the best score.

4. Discard all other aligned segments.

We modified the original SE algorithm slightly as follows. In the original SE, a seed segment was defined as a set of 3 or more consecutive seeds along a diagonal. In the new algorithm, we first label a seed at residue pair *i *and *j *as *tied *if there is another residue pair involving *i *or *j *such that M_*i' j *_- M_*ij *_< 0.5 Å or M_*ij*' _- M_*ij *_< 0.5 Å with a positive SP_*i' j *_or SP_*ij*'_, respectively. Then we define a seed segment as a set of 2 (instead of 3 in the previous version) or more consecutive non-tied seeds. The tied seeds are ignored also during the extension of seed segments to obtain the aligned segments. We made this amendment because we observed instances wherein two, not three, consecutive residues are unambiguously aligned, isolated from other aligned regions. The following steps were newly appended.

5. Extend the surviving *aligned segments *after discarding the inconsistent aligned segments.

6. Change *tied seeds *to *extended pairs *if they do not overlap with already aligned residue pairs.

7. Repeat steps 3 to 5.

The reason for introducing step 5 is that there may be room for extension after removal of inconsistent segments. The additional steps 6 and 7 were used only in the last refinement cycle (see below) to pick up isolated pairs of alignable residue pairs.

For RSE, the sequence alignment by SE without steps 6 and 7 was followed by a rigid body superposition routine KABSCH [27,28]. This two-step process was repeated for up to 10 times until the alignment converged (until the last two alignments were the same). In the rigid-body superposition step, each aligned residue pair was weighted according to the distance *d*_*ij *_between C_α _atoms of the aligned residues: [29], where *d*_0 _and *n *are adjustable parameters with default values of 3.0 Å and 2, respectively. Several combinations of *d*_0 _(= 2.5 to 4.0 Å in 0.5 Å steps) and *n *(= 1 to 4) were tested, but the RSE procedure was rather insensitive to these parameters. During the iteration, the transformation matrix of the superposition that generated the best alignment, in terms of the number of aligned residues, was selected. The final sequence alignment was produced by an additional round of SE that included steps 6 and 7 after two structures were superimposed according to the chosen transformation matrix.

The RSE procedure accepts as input two superimposed structures or two independent structures with a sequence alignment, in which case a superposition is obtained through KABSCH procedure with unit residue weights. In this work, the RSE was run in the latter mode, since some structure alignment programs did not generate superimposed structures. Different programs produced sequence alignments in different formats, which had to be converted into a standard format (the FASTA format). The iterative refinement steps can be skipped by giving *-norefine *command line option, in which case the input superposition is used directly to generate the sequence alignment output. The program is downloadable from the following web site: .

### Reference alignments, structure alignment programs and time measure

We used the CDD (v.2.07) "root node set" introduced in our previous work [12] as the reference sequence alignments with corresponding SCOP domains. We chose this dataset because it is manually procured and because it includes many sequences that are sufficiently dissimilar that structure is needed for their accurate alignment. The 5 'outlier' superfamilies (cd00651, cd01345, cd02156, cd01284, and cd02688) were excluded, for which the CDD alignments were judged questionable as reference alignments [12]. The composition of the dataset is described in Table 1.

We included CE (Algorithm 1.0, Alignment calculator 1.02) [30], DaliLite_2.4.1 [13], LOCK2 [18], FAST [17], MATRAS (version 1.2) [19], MATT [16], SHEBA-4.0 [31], TM-align [20] and VAST (directly from Dr. Gibrat) [14]. We also included SSEARCH from FASTA3 package for pure sequence alignment [32] and SALIGN from Modeller (mod9v6) for profile-profile alignment [33]. The input multiple alignments for SALIGN were prepared from PSI-BLAST alignments (BLASTPGP [34] in blast-2.2.20 package), allowing up to 20 iterations with e-value cutoff of 0.0005 against nr database (as of 04/19/2009). Up to 1,000 sequences with most significant e-values were retained in the multiple sequence alignment. The parameter settings for PSI-BLAST were as described in Marti-Renom et.al. [33]. Otherwise, default values were used for all the programs.

In order to measure the execution times for the methods including the RSE procedure, time-stamps were recorded before and after system calls for the executables. For the CPU times per refinement cycle with CDD alignments, the elapsed time from after the initial structure superposition to the end of refinement cycles, which did not include the file I/O time, was divided by the number of refinement cycles. The CPU times for each alignment were averaged over three independent runs.

## Authors' contributions

BL generated the original idea, CK executed the research, CHT participated in the modification of SE and in the development of the RSE software, and CK and BL wrote the paper. All authors read and approved the final manuscript.
